# Pediatric Flatfeet—A Disease Entity That Demands Greater Attention and Treatment

**DOI:** 10.3389/fped.2020.00019

**Published:** 2020-02-11

**Authors:** Philip J. Bresnahan, Mario A. Juanto

**Affiliations:** ^1^Indian Valley Podiatry Associates, PC, Souderton, PA, United States; ^2^Hospital de Niños Victor J. Vilela, Rosario, Argentina

**Keywords:** pediatric flatfeet, hyperpronation, pes planus, subtalar joint instability, extra-osseous talotarsal stabilization, flat feet

## Abstract

**Background:** Pediatric flatfoot is a common deformity. Unfortunately, the common opinion has been that most children with this faulty foot structure will simply out-grow it, despite no radiographic evidence to support this claim. Every step on a deformed foot leads to excessive tissue strain and further joint damage. Many forms of conservative and surgical treatments have been offered. This study was aimed at investigating the effectiveness of non-surgical and surgical treatment options.

**Main Text:** faulty-foot structure is the leading cause of many secondary orthopedic deformities. A wide range of treatments for pediatric flatfeet have been recommended from the “do-nothing” approach, observation, to irreversible reconstructive surgery. Most forms of conservative care lack evidence of osseous realignment and stability. A conservative surgical option of extra-osseous talotarsal joint stabilization provides patients an effective form of treatment without the complications associated with other irreversible surgical procedures.

**Conclusion:** Pediatric flatfeet should not be ignored or downplayed. The sooner effective treatment is prescribed, the less damage will occur to other parts of the body. When possible, a more conservative corrective procedure should be performed prior to irreversible, joint destructive options.

Pediatric flatfeet are a very common concern for parents and one of the most frequent presenting complaints to a pediatric practice (1). Unfortunately, parents are told that it isn't necessary to treat and to simply ignore this foot deformity. One author even concluded “the best treatment is simply taking enough time to convince the family that no treatment is necessary” (2). Other authors suggest that surgical intervention is rarely necessary, that there is very little evidence for the efficacy of non-surgical care but that non-surgical care should still be considered (2–4). While a child's foot shape and size change over the first several years of life, there is a recognizable difference between normal development and pathology at any age. Foot and ankle specialists know that sooner or later, flat and misaligned feet will slowly lead to other pathologies within the foot and ankle and proximal structures (5).

Walking is one of the most common unconscious functions of our body. We are told to take 10,000 steps a day, to keep healthy (6). The stability and alignment of the foot is very important because the foot is the foundation to the upright body. Failure to identify and treat foot and ankle instability and misalignment will lead to long-term tissue pathology. Foot misalignment has been shown to reduce propulsive phase push off muscle power by 35%, which seems to be an obvious gait inefficiency (7). Tissue strain leads to pain, increased deformity, and eventually decreased activity level. The body's metabolic rate decreases as a result which leads to other health risks such as obesity, diabetes, hypertension, and heart disease (8, 9).

The goal in treating any disease is early identification and intervention with a method proven to achieve the desired outcome that is measurable and makes sense. There is a long evolution in the internal correction of this faulty-foot alignment disease. The use of extra-articular realignment and stabilization techniques have stood the test of time. The medical necessity and evidence basis of extra-osseous talotarsal stabilization has been established (10). The time has come to embrace this minimally invasive, reversible solution as an early intervention to pediatric flatfeet.

## Hindfoot Alignment and Biomechanics

**Figure 1:**
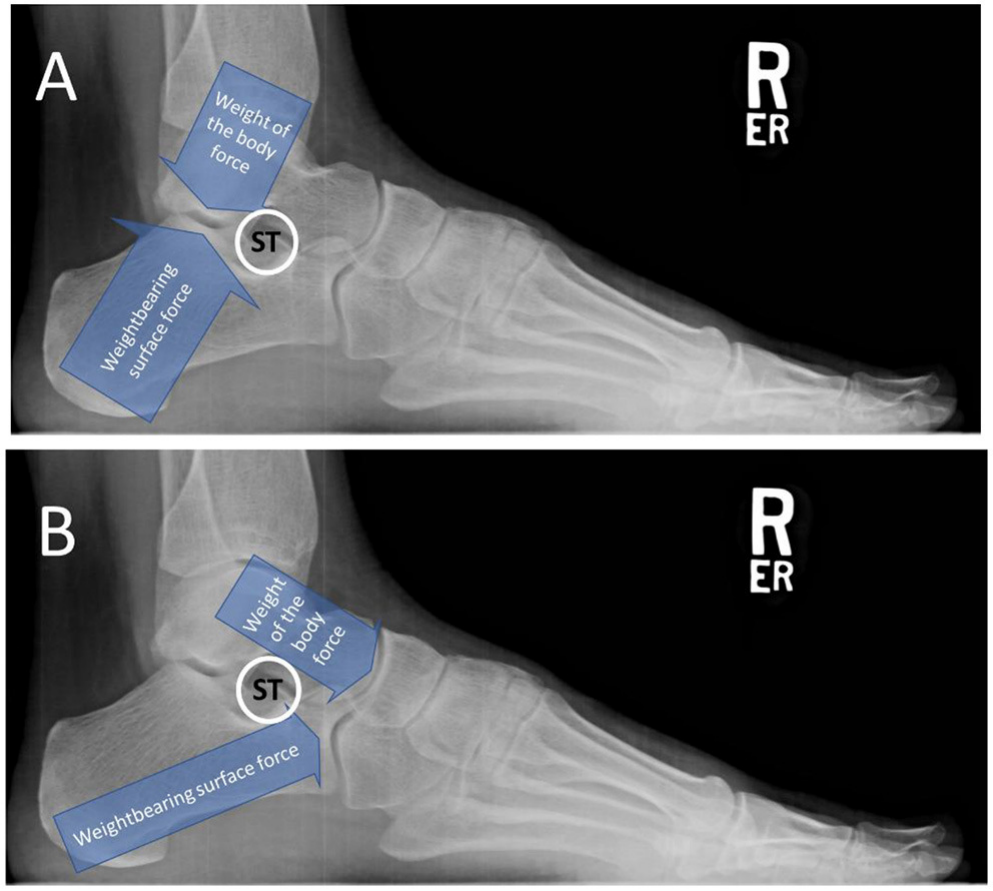
Weightbearing lateral foot radiograph of a foot in resting stance position showing normal talotarsal joint forces. **(A)** Forces acting posterior to the sinus tarsi at heel strike. The sinus tarsi is labeled as ST. **(B)** Forces acting anterior/distal to the sinus tarsi as the plantar surface of the foot makes contact with the weightbearing surface. Sinus tarsi should remain open.

The talus is an osseous extension of the leg that articulates distally with the hindfoot bones. The distal and plantar articular facets of the talus interact with the calcaneus and navicular to form the talotarsal joint (TTJ). The large posterior talocalcaneal joint is responsible to accept a vertical force as much as 80% at heel strike that reduces to slightly greater than 50% of the weightbearing forces at mid-stance (11, 12). These forces occur posterior to the posterior aspect of the sinus tarsi (Figure 1A). The remaining force acts distal to the anterior aspect of the sinus tarsi (Figure 1B). The balance of the forces acting within the TTJ is responsible for an efficient hindfoot machine by performing the essential function of converting vertical force into a horizontal force.

**Figure 2:**
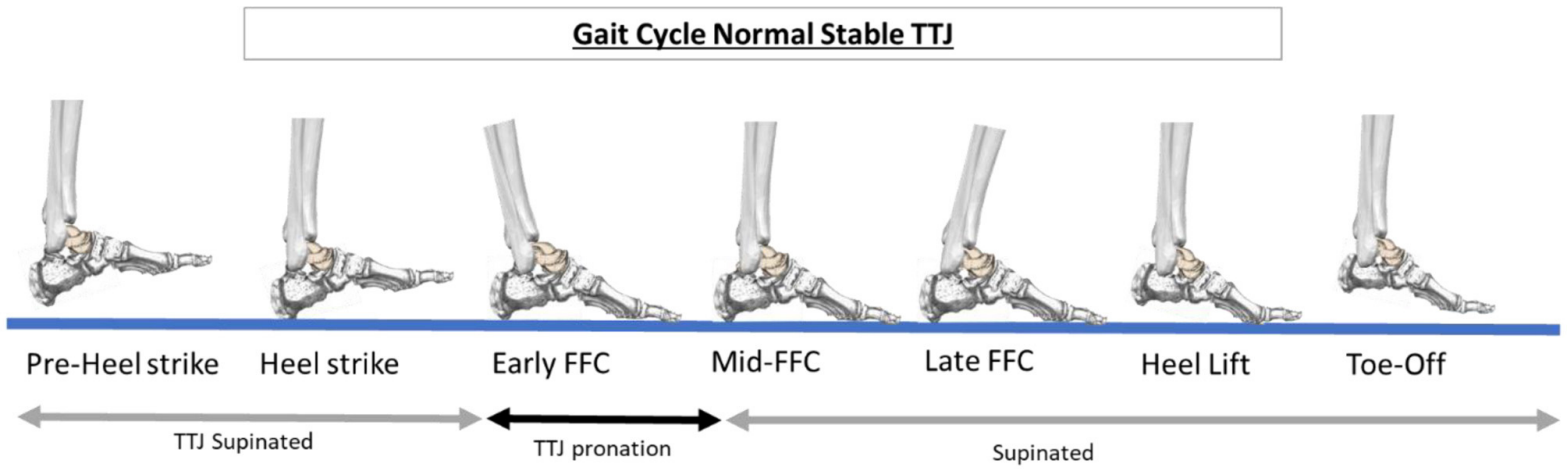
Schematic showing the portions of the gait cycle with a normal stable talotarsal joint (TTJ). It also shows when the TTJ should be in a supinated or pronated alignment. “FFC” is full plantar foot contact.

The TTJ allows for the locking and unlocking of the joints within the medial column of the foot during weightbearing. TTJ pronation unlocks the joints which provides the adaptability of an uneven weightbearing surface at the beginning of the full, plantar foot contact portion of the gait cycle. The TTJ should resupinate at approximately 1/4th to 1/3rd of the full-foot contact (Figure 2). This strengthens the foot structure as it prepares for heel lift and forward foot propulsion.

**Figure 3:**
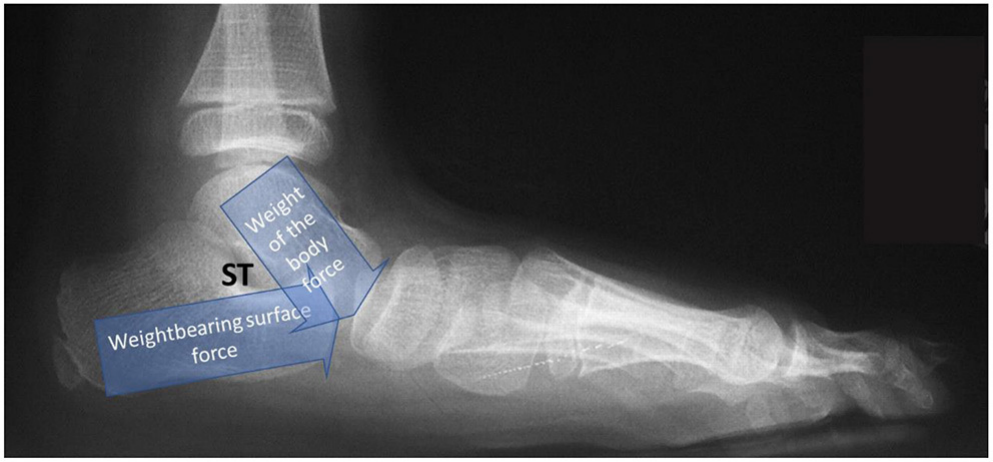
Weightbearing lateral foot radiograph of a foot in resting stance position showing partial dislocation of the talotarsal joint illustrating joint forces. Loss of TTJ alignment occurs at the initial stage of full plantar foot contact. Sinus tarsi is obliterated. Excessive forces are thrust on the mid-foot.

The primary component of a flexible “flatfoot” is the loss of stability and alignment of the TTJ (Figure 3) (13). A flatfoot cannot occur unless the talus and calcaneus are malaligned. Instability of the TTJ results in the pathologic redistribution of the weightbearing forces acting within the hind-, mid-, and forefoot. The forces are non-existent during the swing phase and excessive during the weightbearing stance phase of the gait cycle. TTJ displacement leads to osseous malalignment, increased strain to ligaments, and increased muscle-tendon contraction until the foot has left the weightbearing surface (14). This cascade of excessive tissue loading and unloading of forces occurs with every step taken.

**Figure 4:**
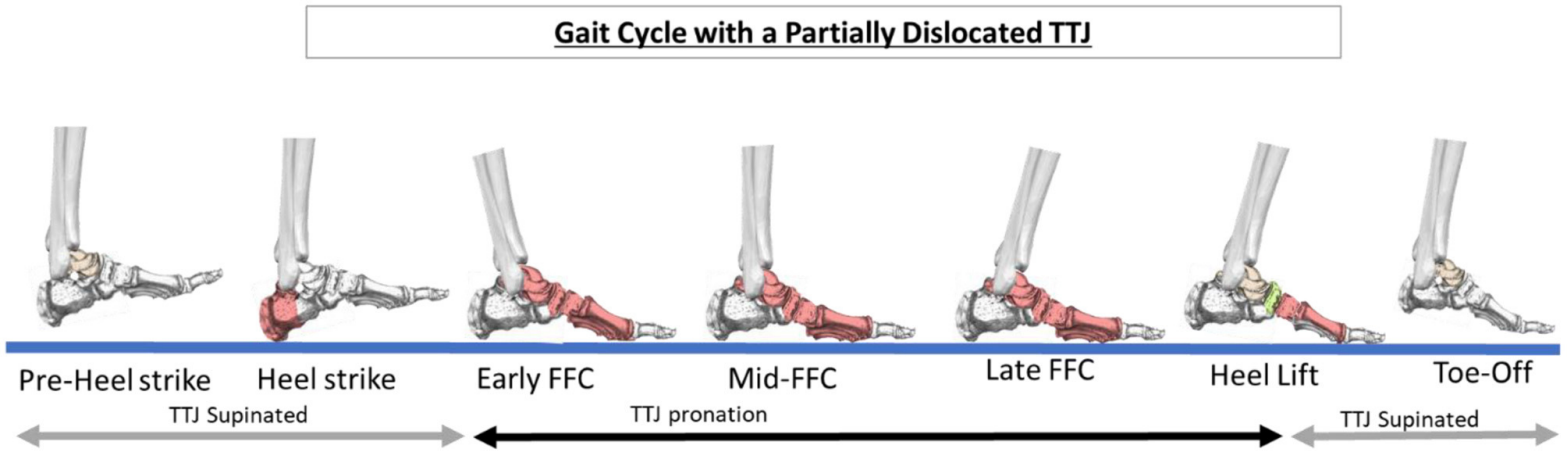
Schematic showing the portions of the gait cycle with an unstable, partially dislocated talotarsal joint (TTJ). The TTJ remains in a pronated, unlocked position longer than normal. “FFC” is full plantar foot contact. The joints and soft tissues will be forced to compensate for the excessive forces acting on the medial column of the foot.

**Figure 5:**
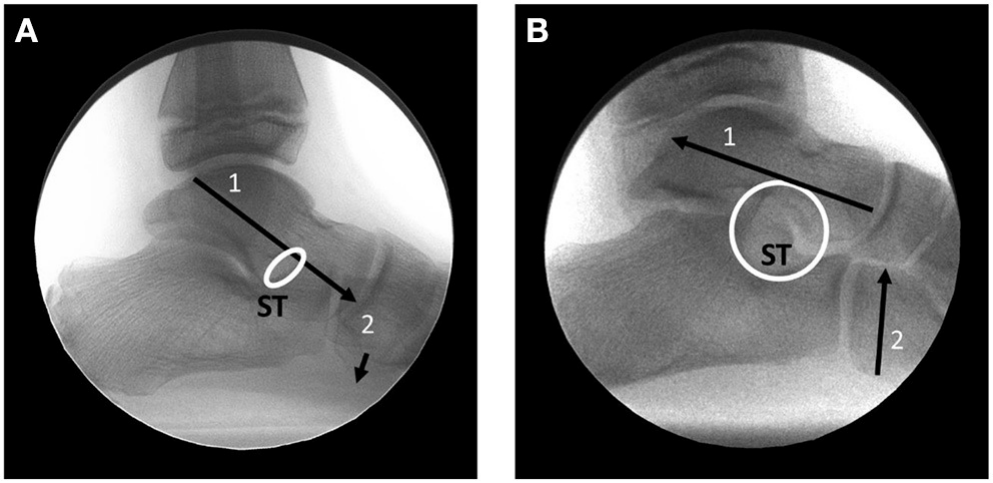
Lateral weight bearing fluoroscopic radiographs. **(A)** Relaxed stance position. The talus has partially dislocated on the calcaneus (1). The sinus tarsi (ST) is obliterated and the navicular has dropped (2). **(B)** The same foot with the articular facets of the talus repositioned on the calcaneus (1). Sinus tarsi (ST) is re-opened and the navicular is elevated (2).

A visible sign of TTJ misalignment is the lowering of the medial arch of the foot, contributing to a flatfoot. That is because the joints of the medial arch are unlocked longer than they should during the weightbearing portion of the gait cycle leading to excessive pronation (Figure 4) (15). It is navicular drop/sag that leads to the lowering of the medial arch of the foot (16, 17). The navicular is forced plantar-medially because of subtalar joint (STJ) instability—specifically the talus partially dislocating on the calcaneus. Realignment of the STJ shows the restoration of the elevation of the navicular (Figure 5).

**Figure 6:**
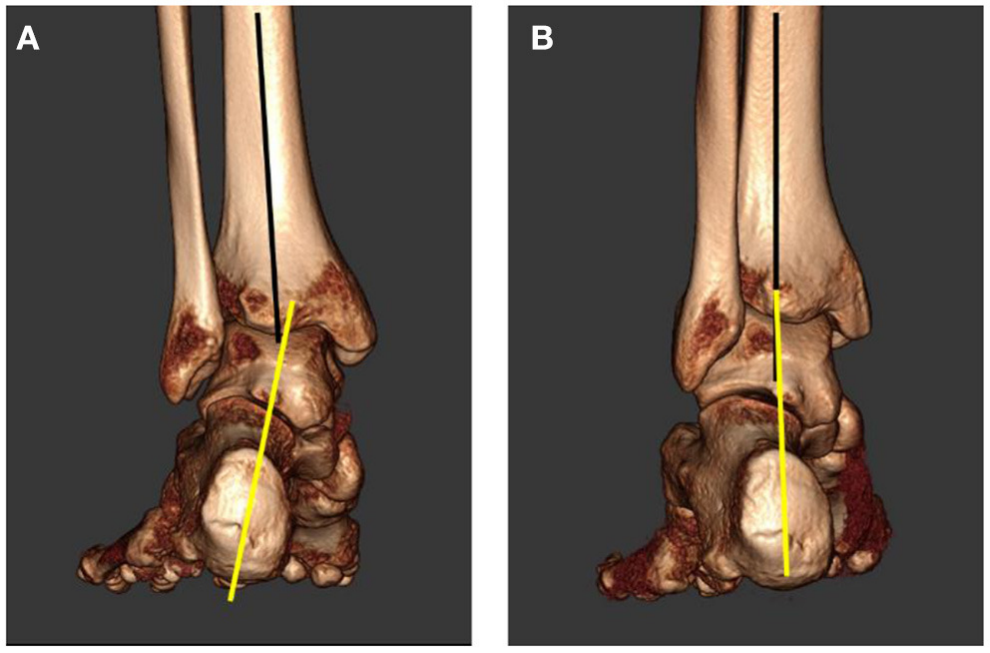
Posterior 3-dimensional weightbearing computed tomography of the hindfoot. **(A)** Hindfoot is in relaxed-stance position, notice the heel valgus (yellow line). **(B)** Same foot with the subtalar joint placed into neutral alignment, notice the rectus heel alignment.

Some feet may also exhibit a calcaneal valgus, but this is not always present with TTJ instability (18, 19). Heel valgus occurs as a form of compensation from excessive internal talar rotation and plantar flexion (Figure 6) (20). This is a very important consideration because surgeons may choose to cut and shift the heel bone to a more “aligned” position, but the excessive talar instability will still be present, unaffected by this form of treatment.

## Diagnosis of a Flatfoot

The diagnosis of a flatfoot has historically been determined by many different factors. This could be one of the many reasons why there is so much confusion in the diagnosis and subsequentially, the treatment of flatfeet. There are subjective, clinical observations that must be confirmed by objective, standardized, and validated radiographic angular measurements. A major issue with clinical observations, such as arch height, heel eversion, and plantar foot prints, is that they are subjective and poorly reproducible from examiner to examiner. These observations are unable to pin-point a specific anatomic consideration, such as internal osseous malalignment.

Resting- or relaxed-stance position weightbearing radiographs should be the gold standard in the diagnosis of a flatfoot. Osseous malalignment leads to faulty foot structure and the only way to visualize foot bone alignment is with radiographs taken while the individual is standing on their feet. There are specific validated radiographic angles that show normal or abnormal alignment of the osseous foot structures. The dorsoplantar (DP) view measurements include the talar second metatarsal along with the talar navicular head uncovering. Key features on the lateral radiograph include the opening or obliteration of the sinus tarsi, talar declination angle, talar first metatarsal angle, calcaneal inclination or pitch angle, and navicular position (Table 1).

**Table 1:** Comparison of normal to abnormal radiographic findings.

**Radiographic angle**	**Normal range**	**Abnormal range**
Talar second metatarsal (DP)	<16	>16
Talar head uncovering (DP)	<7	>7
Calcaneal pitch/inclination (Lateral)	20 to 30	<20
Sinus tarsi (Lateral)	Open	Partial/Full Obliteration
Talar declination (Lateral)	<21	>21
Talar first metatarsal (Meary's angle) lateral	0	>4
Navicular position (Lateral)	Plantar aspect is dorsal to the horizontal bisection of the cuboid	Plantar aspect of the navicular is plantar to the horizontal bisection of the cuboid
Calcaneal axial (Posterior)	Rectus alignment	Valgus alignment

The diagnosis of a “flatfoot” is too generic. It does not give an accurate indication of what is “broken.” The specific “broken” components leading to the overall less-than-aligned foot should be individually described to patients. A recurrent talotarsal joint dislocation (RTTJD) is the primary deformity of a flexible “flatfoot.” RTTJD indicates that there is a condition where the TTJ has lost its normal alignment during weightbearing throughout the gait cycle. RTTJD is a “flexible” verse rigid pathology and important in determining the best form of treatment. Realignment of the TTJ results in an increase in arch height, re-opening of the sinus tarsi, normalization of joint forces, increased navicular height, normalization of the cyma line, rectus calcaneus, and possibly normalization of the calcaneal inclination angle.

Some clinicians will assume a foot to be “normal” unless there is complete loss of the medial arch (21). RTTJD can exist with a “normal” appearing arch, due to a normal calcaneal inclination angle and a navicular that does not drop, but it is still considered a pathological phenomenon. A rigid, non-reducible flatfoot still leads to a prolonged degree of pronation during the gait cycle. All of these factors lead to further confusion in the diagnosis abnormal foot alignment. Other disease processes such as a tarsal coalition must also be ruled out.

## The Myth of “Autocorrection”

One of the reasons why many pediatricians downplay the severity of this orthopedic deformity is that there are claims the flatfoot self-repairs. The evidence basis of these claims is based on a cross-sectional epidemiologic study by determining the heel-to-arch width ratio in two different age groups (22). This was not a longitudinal study over an 8-year period. The research showed that 97% of children 2 years or younger had a flatfoot according to the foot-print ratio and that the ratio decreased to 4% when measured at a separate group of children 10 years old. The assumption was that the majority of flatfeet would resolve by age 10. This data only evaluated the plantar fat pad of the medial arch, it did not take into consideration the osseous alignment. The results of the comparison lack the necessary data to conclude auto-correction of a faulty foot structure.

How could a misaligned, unstable foot become internally realigned and stabilized when excessive abnormal forces are acting on those structures with every step taken? Osseous malaligned structures do not realign. Juvenile hallux abductovalgus deformities, for example, become progressively worse, not better (23). There are no long-term follow up radiographic studies that document osseous realignment of a pediatric flatfoot (24). The non-surgical treatment of flatfeet has been studied and shown to be ineffective in addressing the osseous misaligned, so how could anyone expect the feet to auto-heal? There is documentation that adult flatfeet continue to progressively get worse (25, 26). Those asymptomatic pediatric flatfeet eventually lead to a symptomatic adult flatfoot.

## Outcome if not Treated

If you don't fix what is broken, it remains broken. If forces continue to act excessively on tissues, those tissues will create a defense mechanism in an attempt to handle those excessive forces as described by Wolff and Davis (27, 28). Eventually, a critical threshold is reached when the tissues can no longer compensate, at that point tissue failure occurs. Subsequentially, joint and other tissues will be forced to compensate for the loss of the primary stabilizing tissues. Excessive force and pressure within the joint leads to an inflammatory reaction. If those repetitive excessive forces remain unchecked, chronic inflammation will result leading to arthritis and joint destruction.

Hindfoot joint displacement has been found to be the most common finding associated with the onset of symptoms (29). Hyperpronation is named at the leading etiologic factor to many chronic foot pathologies such as plantar fasciopathy (30–32), posterior tibial tendon insufficiency (33–37), first ray deformities (38–40), hallux limitus (41, 42), tarsal tunnel syndrome (43, 44), and posterior tibial tendon dysfunction (45, 46). Proximal manifestations attributed to hindfoot misalignment are ankle joint pathology (47, 48), growing pains, medial tibial stress syndrome/shin splints (49–51), knee pathology (52–57), hip pathology (58, 59), pelvic tilt (60), and even back misalignment (61, 62).

There are additional considerations that must be also factored into this disease process. Children are taken to a pediatrician or foot care specialist and are told they have a musculoskeletal deformity, they are deformed. The physician is tries to “convince the parent” that there is no need for treatment? This will create a psychological disturbance in the child's mind. There is also the issue of decreased physical activity in sports. Competitive sports are a big part of lower and upper school. Children who are less competitive are looked down upon against their more athletic classmates.

The health-related quality of life is significantly impaired in children with flexible flatfoot (63). Children with flexible flatfeet have an increased body mass index (BMI) compared to children with normal appearing feet (64). One of the primary treatments of obesity is to increase the metabolism to burn off those calories (65). Walking on misaligned feet is less-than-ideal as muscles will have to work harder to lift the inner arch and sooner rather than later, pain will ensue stopping the weightbearing activity (66, 67).

## Primary Goals of Treatment

The successful treatment of flatfoot must take into account the underlying pathology to optimize outcomes. We must be mindful of the three principles of orthopedic treatment. That which is crooked, make straight. That which is unstable, make stable. Joint preservation should be attempted over joint-destructive measures, when possible. The primary deformity of a flatfoot is RTTJD so the goal of treatment should be aimed at realigning the articular facets of the TTJ and to maintain that alignment, while still allowing a natural range of motion. When considering the various treatment options for flatfeet, we must ask the question, does that treatment make sense? Is it capable of accomplishing the primary goals of treatment? If it doesn't meet the standards, then should it be considered below the standard of care to offer that form of treatment?

## Treatment in the Absence of Pain

Much of the published literature on pediatric flatfoot advises that treatment is only necessary when pain is present. This goes against the principles of medical ethics. Should we wait until a diabetic patient falls into a hyperglycemic coma to begin care? Should we wait until someone has their first heart attack to begin treatment? Of course not. The same is true when considering the treatment of malaligned feet. Sooner or later certain tissues will no longer be able to handle the excessive forces and that tissue will partially rupture. If no intervention is provided to decrease the strain on the tissue it will eventually result in a complete rupture. If there is an imbalance of force acting within a joint, eventually those excessive forces will have a negative impact on the cartilage until the cartilage is damaged. If the forces aren't normalized, that damage becomes chronic eventually leading to irreversible changes. It makes sense therefore to eliminate the tissue strain to prevent the partial rupture, to balance the joint facets to prevent arthritis, and to improve the biomechanics of the foot.

It is also important to make a connection between pain elsewhere in the body and other symptoms related to a faulty foot structure. It is true that many pediatric patients, or even adult patients, with flat feet have no pain or symptoms within their feet. However, those symptoms can show up as pain to other parts of the body as previously discussed, i.e., growing pains, shin splints, knee pain, hip pain, back pain, and other functional symptoms. Ultimately, the decision on accepting a treatment has to be made by the parents or legal guardian.

## Forms of Non-surgical Treatment

There have been many non-surgical forms of treatments prescribed for pediatric flatfeet including activity modification, orthopedic shoes, foot orthoses, stretching and strengthening exercises, and non-steroidal anti-inflammatory medications.

### Observation

The most common form of treatment is simply to ignore this orthopedic disease as a normal variant. This “non-treatment” should be considered below the standard of care as multiple studies have identified flat/over-pronating feet are a significant contributing factor to the development and progression of pathologies within the foot, knee, hip, and back pain (47, 50, 53, 62, 68–70). Tens of millions of steps taken on malaligned feet will eventually take their toll.

### Physical Therapy/Muscle Strengthening

Muscle strengthening exercises have been strongly recommended. There is very limited, if any, scientific basis for this form of treatment. Does it even make sense? The strong muscles of the lower extremity are over-powered by the collapse of the medial arch. It has been shown that the pronatory muscles are already contracting to lift the arch and assist in re-supinating the foot (37, 71). Another study found that the posterior tibial tendon is incapable of fully accommodating hindfoot misalignment (72). This is why there are no long-term studies showing a positive effect or correction to a flatfoot and that this is a less-than-ideal long-term solution.

### Arch Supports/Foot Orthoses

Use of arch supports has long been advocated even though there is little to no evidence basis (73, 74). There are very short-term positive results in reduction of symptoms but longer follow up is lacking. Staheli concluded “Treatment of children with physiologic flatfoot with orthoses or shoe modifications not only is ineffective but is uncomfortable and embarrassing for the child and is associated with lowered self-esteem in adult life” (4). A prospective study was undertaken to determine whether flexible flatfoot in children can be influenced by corrective shoes and inserts (75). They concluded “that wearing corrective shoes or inserts for 3 years does not influence the course of flexible flatfoot in children.” Many other studies have discovered similar inability of custom foot orthotics or specialized shoes to be ineffective in the treatment of pediatric flatfoot (24, 76). Furthermore, other studies have shown that there is no radiologic improvement with the use of arch supports/custom foot orthotics (77). Arch supports/foot orthoses should be considered a subtherapeutic form of treatment.

## Non-Conservative Surgical Treatment

There are advocated surgical procedures to realign and stabilize the hind- and mid-foot. These osseous procedures such as a lateral calcaneal lengthening, opening cuneiform osteotomy, navicular lifting procedures, can be associated with many risks and potential complications such as sural nerve injury, surgical wound dehiscence, under or over-correction, and graft subsidence (78). Due to the invasiveness of these surgical options, it was recommended to delay this form of treatment after failure of any other potential option that can accomplish the treatment goals.

## Conservative Surgical Treatment

**Figure 7:**
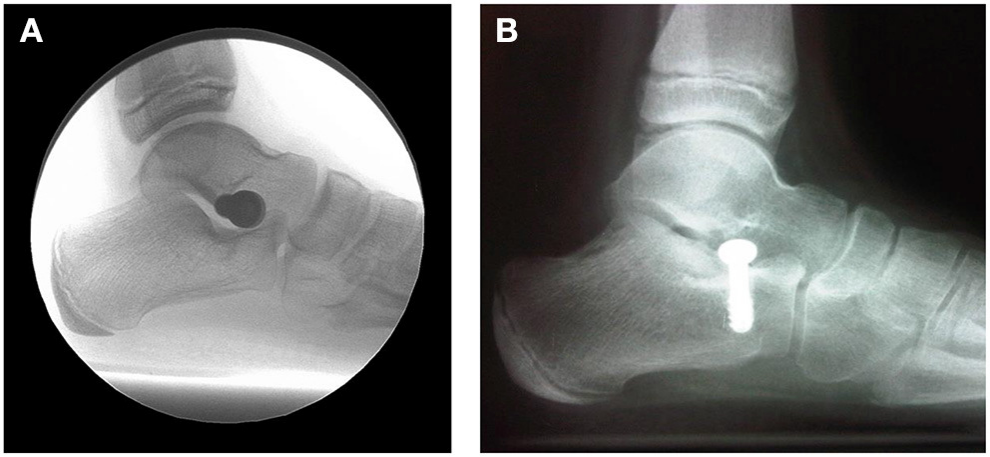
**(A)** Lateral weightbearing radiograph of an extra-osseous sinus tarsi implant. **(B)** Intra-osseous screw acting to stabilize the talotarsal joint.

The insertion of a stent into the sinus tarsi has been advocated and performed by foot and ankle surgeons for many decades (79–82). This procedure was born out the necessity to achieve the desired results of foot realignment without sacrificing joint structures. Over the past many decades many different designs of implants and materials have evolved into three types of methods. Intra-osseous talotarsal stabilization (Figure 7A) involves the partial insertion of a screw type implant into either the floor of the sinus tarsi of the calcaneus (83, 84), or partially into the lateral process of the talus (85). This method is a true joint blocking—arthroereisis. Many surgeons simply use a rather inexpensive large orthopedic screw. This technique is limited to patients 12 to 18 years of age. Typically, the screw is removed within 18 months of insertion. Correction is achieved while the screw is *in situ* but the long-term results after screw removal are unknown.

**Figure 8:**
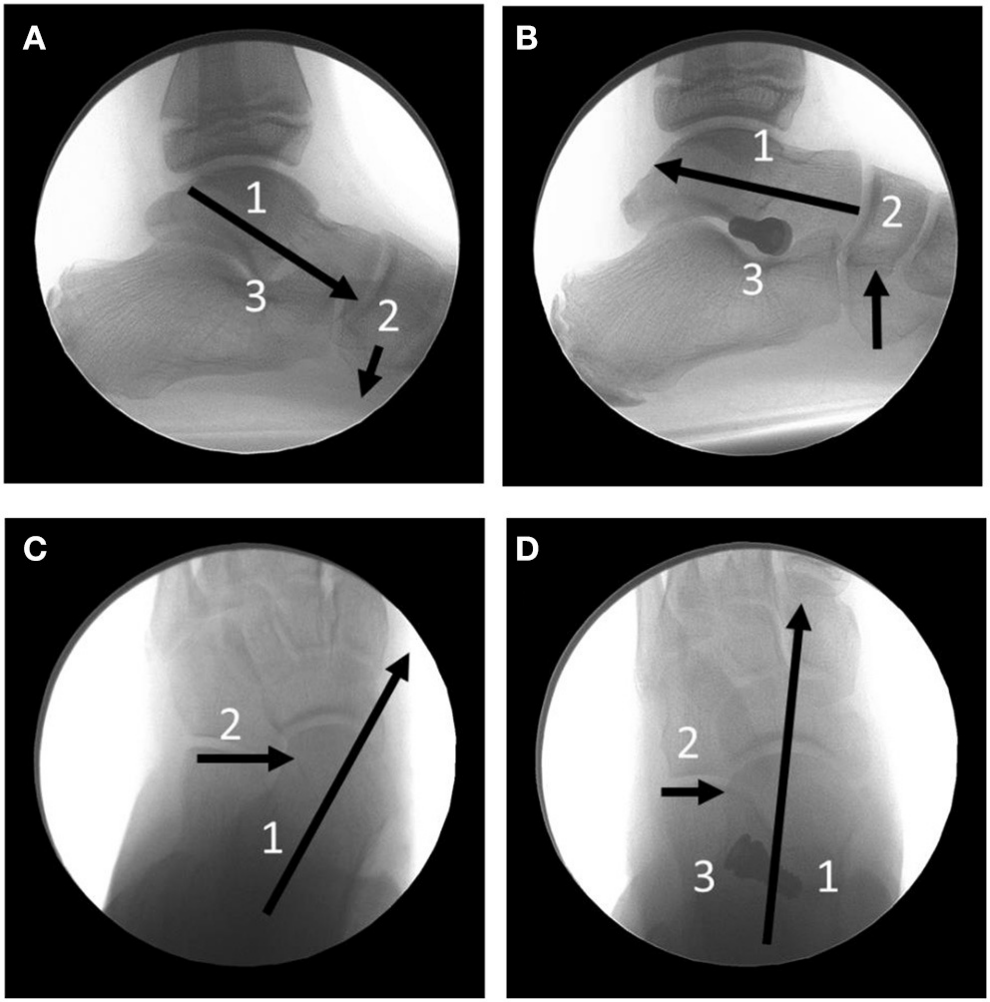
Full Weightbearing, relaxed stance position fluoroscopic radiographs. **(A)** Lateral image showing the anterioplantar parital dislocation of the talus (1) on the calcaneus. The navicular (2) is forced plantarly. The sinus tarsi (3) is obliterated. **(B)** The same foot post-extraosseous talotarsal stabilization. Notice the realignment of the talus on the calcaneus (1) and elevated navicular (2). (3) is the HyProCure (GraMedica, Michigan, USA) sinus tarsi implant. **(C)** Dorsoplantar view of the hindfoot. The talus (1) is medially and anteriorly displaced on the calcaneus (2). **(D)** The same foot post-extraosseous talotarsal stabilization. Notice the lateralization of the talus (1) and reduction of the anteriomedial displacement of the talus (2). (3) The HyProCure implant.

Extra-osseous sinus tarsi stents (EOTTS) (Figure 7B) are the second type of devices inserted to maintain the alignment and stability of the TTJ. During weightbearing the anterior process of the talus pushes against the implant until the anterioplantar surface of the implant comes into contact with the posterior surface of the anterior floor of the sinus tarsi, formed by the calcaneus. These devices can correct in both the transverse and sagittal, and therefore frontal planes (Figure 8).

There are numerous studies that have provided the evidence basis, safety and effectiveness of this minimally invasive joint sparing procedure. There is evidence of radiographic normalization of osseous realignment (86, 87), restoration of navicular height/position (88), maintenance of arch height (89, 90), restoration of joint congruity (3), rebalancing of subtalar joint forces (91), decreased strain to the plantar fascia (92), posterior tibial nerve (93), and tendon (94), improved function and pain scores (95), improved ankle joint alignment (96), return to sports activity, and improved emotional status (97). Pediatric patients report a high degrees of satisfaction, and their quality of life was improved and not compromised by the procedure (98, 99).

Complication of extraosseous sinus tarsi implants include pain within the sinus tarsi area, device extrusion/displacement, and under/over-correction. These less-than-ideal situation are self-resolving once the procedure is revised or with permanent removal of the implant. There have been no long-term complications as a result of the stent being in the sinus tarsi. A benefit-risk analysis will show the benefits far exceeds any potential risks. Other less-conservative surgical procedures can still be performed.

## Conclusion

Pediatric flatfoot is a progressive mechanical disease process that is linked to many other musculoskeletal, metabolic, and physiological pathologies. There is no supportive evidence that osseous misaligned structures will auto-correct. These misaligned feet do not get better, they progressively worsen. Failure to address this disease process will lead to deleterious effects to many parts of the body and to one's physical and mental health. Foot pain may not be present in most people with flatfeet however, the symptoms of flatfeet can appear as knee, hip, and back pain. The algorithm for the treatment of flatfeet must begin with measures proven to realign and stabilize the malaligned osseous structures, i.e., the talotarsal joint, while still allowing a natural range of motion, without compromising adjacent joint structures.

We must acknowledge that there is no evidence of auto-correction of pediatric flatfeet. That this orthopedic disease will lead to other orthopedic, metabolic, and even mental distress if not properly addressed. Conservative options are proven to be ineffective in the realignment of the osseous pathologies. Traditional osteotomies and arthrodesis procedures have many known complications and risks. EOTTS is superior option to external measures in realigning and stabilizing the TTJ. EOTTS should be considered as a minimally invasive, conservative surgical option prior to irreversible osseous surgery, when indicated. EOTTS can be used in combination with other treatment options.

## Author Contributions

All authors listed have made a substantial, direct and intellectual contribution to the work, and approved it for publication.

### Conflict of Interest

PB has no financial interest in Gramedica, but has been reimbursed for travel expenses to speak at seminars (as all speakers are), but received no honoraria while discussing the type of procedure performed in the article. MJ is listed as a clinical instructor for the Graham International Implant Institute. Reviewer MG is the founder and president of the Graham International Implant Institute, and the company GraMedica that produces the HyProCure® implant, which is used to treat bone misalignment in flat feet.
