# Corrigendum: Pediatric Flatfeet—A Disease Entity That Demands Greater Attention and Treatment

**DOI:** 10.3389/fped.2021.735481

**Published:** 2021-09-01

**Authors:** Philip J. Bresnahan, Mario A. Juanto

**Affiliations:** ^1^Indian Valley Podiatry Associates, PC, Souderton, PA, United States; ^2^Hospital de Niños Victor J. Vilela, Rosario, Argentina

**Keywords:** pediatric flatfeet, hyperpronation, pes planus, subtalar joint instability, extra-osseous talotarsal stabilization, flat feet

In the original article, we neglected to include a conflict of interest statement (see below).

PB has no financial interest in Gramedica, but has been reimbursed for travel expenses to speak at seminars (as all speakers are), but received no honoraria while discussing the type of procedure performed in the article. MJ is listed as a clinical instructor for the Graham International Implant Institute.

Reviewer MG is the founder and president of the Graham International Implant Institute, and the company GraMedica that produces the HyProCure® implant, which is used to treat bone misalignment in flat feet.

The authors apologize for this error and state that this does not change the scientific conclusions of the article in any way. The original article has been updated.

## Publisher's Note

All claims expressed in this article are solely those of the authors and do not necessarily represent those of their affiliated organizations, or those of the publisher, the editors and the reviewers. Any product that may be evaluated in this article, or claim that may be made by its manufacturer, is not guaranteed or endorsed by the publisher.

